# Validation of quantitative polymerase chain reaction with Southern blot method for telomere length analysis

**DOI:** 10.4155/fsoa-2017-0115

**Published:** 2018-01-18

**Authors:** Mohamad Tarik, Lakshmy Ramakrishnan, Harshpal S Sachdev, Nikhil Tandon, Ambuj Roy, Santosh K Bhargava, Ravindra M Pandey

**Affiliations:** 1 Department of Cardiac Biochemistry, All India Institute of Medical Science, New Delhi, India; 2 Department of Pediatrics & Epidemiology, Sitaram Bhartia Institute of Science & Research, New Delhi, India; 3 Department of Endocrinology & Metabolism, All India Institute of Medical Science, New Delhi, India; 4 Department of Cardiology, All India Institute of Medical Science, New Delhi, India; 5 Department of Pediatrics, Sunderlal Jain Hospital, New Delhi, India; 6 Department of Biostatistics, All India Institute of Medical Science, New Delhi, India

**Keywords:** correlation, MMqPCR, qPCR, reliability, reproducibility, Southern blot, telomere biology

## Abstract

**Aim:**

Telomere length (TL) measurement by quantitative polymerase chain reaction (PCR) has been widely accepted, but limited information regarding its validation with a gold-standard technique is available.

**Materials & methods:**

We measured TL by Southern blot and monochrome multiplex quantitative PCR (MMqPCR) and validated the results of TL in leukocytes of 94 participants with mean age 43.2 years, BMI 19–41 (mean 27.8 ± 4.3) kg/m^2^.

**Results:**

A significant positive correlation was observed between TL measured by MMqPCR and Southern blot assay (correlation coefficient r = +0.896, p < 0.0001). The inter- and intra-assay CVs of the MMqPCR assay were 5.3 and 4.07%, respectively.

**Conclusion:**

We observed that experimental discrepancies have an impact on TL analysis and there is a need to improve the optimum conditions.

## Lay abstract

The aim of this study was to validate the relative telomere length measurements by different methods. We measured leukocyte telomere lengths by Southern blot and monochrome multiplex quantitative polymerase chain reaction in 94 participants and a strong correlation was shown between both techniques. The results of our study suggest that the monochrome multiplex quantitative polymerase chain reaction method can be employed reliably for measurement of telomere length in epidemiological studies involving a large number of samples.

Telomeres are tandemly repeated hexa-nucleotide sequence at the ends of chromosomes that protect the coding DNA by capping chromosomal ends and together with associated proteins. This nucleotide sequence (5′-*TTAGGG*-3′, in human) repeats approximately 2500-times but shows intraindividual variation and differs with different stages of growth. Telomeres become shorter with every somatic cell division due to incomplete DNA replication, resulting in shortening of telomeric DNA up to 20–200 bp with each cell division. The gradual loss of telomeric DNA contributes to the essential cellular senescence and programmed cell death which is associated with normal cellular aging [1]. Lifestyle factors can modulate telomere length (TL). The accelerated telomere attrition is associated with increased risk for several age-related diseases such as cardiovascular disease [2–5], diabetes [6] and Alzheimer's dementia [7]. They also play an important role in the progression of cancer. However due to large interindividual variability and lack of standardization of measurement methods, usage of TL measurement in clinical settings is limited.

Different techniques have been employed to determine telomere length and include Southern blot analysis [1,8], quantitative polymerase chain reaction (PCR) [9,10], flow-fluorescence *in situ* hybridization (FISH) [11] and single telomere length analysis method [12]. Southern blot technique measures absolute length of telomere restriction fragments (TRF) and is the gold standard method. Usage of this technique is limited in studies involving large number of samples due to the method being time consuming, costly, labor intensive and requiring high concentration of DNA. The single telomere length analysis method is labor intensive and is again not suitable for testing large number of samples. The flow-FISH method is used to assess TL in subsets of cells separated from fresh blood samples and requires expensive equipment which may not be available in many laboratories and cannot be used to assess TL in tissues and stored samples. The quantitative PCR technique which gives relative TL in short time and requires small amount of DNA (20 ng per reaction) is widely used in epidemiological studies. This technique was introduced by RM Cawthon in 2002 [9]. The method entails detection of telomeric DNA with fluorescent signals (T) using partially mismatched primers in a 96-well format on real time-quantitative PCR (qPCR) platform. The telomeric DNA measurement is normalized with a single-copy housekeeping gene (S) amplified in same sample in a different plate and T/S ratio is computed which is a measure of relative TL. An improved version of this technique monochrome multiplex qPCR (MMqPCR) was established by the same author in which both telomeric DNA and single-copy gene are amplified in a same well of a plate and shows less variability compared with monoplex qPCR and has lesser sample requirement [10]. Studies have reported wide range of CVs (2–28%) for measurement of TL by qPCR suggesting that reproducibility is a concern with qPCR [13,14]. Proper optimization of qPCR conditions is of importance to reduce variability [15]. Comparative studies for validation of qPCR with the gold standard Southern blot technique are limited. In the current study we compared the relative TL measured by multiplex quantitative PCR with absolute TL measured by Southern blot technique.

## Materials & methods/experimental

**Figure 1.**
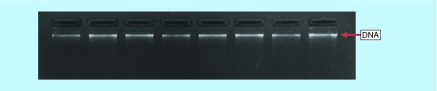
**Visualization of genomic DNA on 0.8% agarose gel.** A single band of good intensity shows a good quality and integrity of the genomic DNA.

Leukocyte TL measurement was performed in 94 whole blood samples collected in anticoagulated EDTA tube from participants of New Delhi Birth Cohort Study after taking consent. Ethical clearance was obtained from institutional ethical committee. DNA was isolated from whole blood using DNeasy Blood & Tissue Kit (Cat. No: 69506, Qiagen, Germany) and was quantified using NanoDrop spectrophotometer (Thermo Fisher Scientific, MA, USA). The ratio of 260/280 and 260/230 absorbance are determined to assess the purity of the sample. DNA integrity was checked by running genomic DNA on 0.8% agarose gel at 2.5 volt/cm for 1 h at room temperature (Figure 1).

### Measurement of TL by qPCR

**Figure 2.**
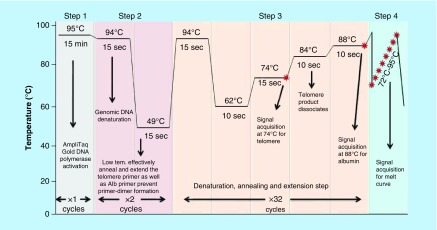
**Thermal cycling protocol for monochrome multiplex quantitative polymerase chain reaction on Real-Time PCR machine (IQ5, Bio-Rad).** In the first step, Taq DNA polymerase was activated and genomic DNA was denatured. In second step, primers were annealed and extended effectively at low temperature in two cycles. In third step, the cycles were repeated. The acquisition at 74°C provided the Ct values for telomeres and the acquisition at 88°C provided the Ct values for the single-copy gene (*albumin*). In the final step, melt curve was prepared by increasing temperature at the end of the experiment. Tem.: Temperature.

**Figure 3.**
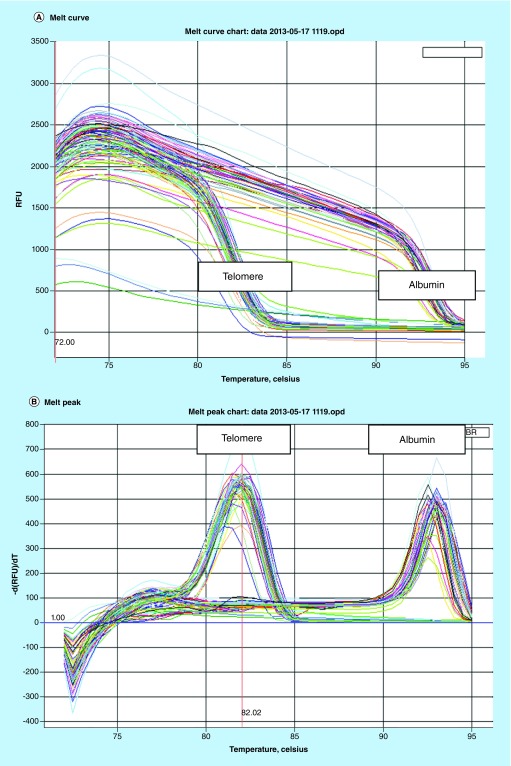

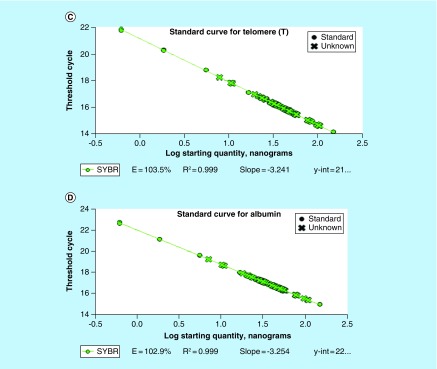
**Melt curve and melt peak and standard curve of telomere and *albumin* gene.** To prepare melting curves, reactions were cooled to 72°C after the completion of step 3 of thermal cycling and signals were acquired from 72 to 95°C, in 0.5°C steps, with a 30 s dwell period per step. The melt curve **(A)** and melt peak **(B)** show good homogeneity and specificity of amplified product of telomere and *albumin*. In the semi log plot of DNA concentration versus cycle threshold, both standard curves **(C & D)** were linear over the 81-fold DNA concentration range of standard DNA (R^2^ = 0.999). The efficiency and slope of standard curves of telomere (E = 103.5%, slope = -3.241) and *albumin* (E = 102.9%, slope = -3.254) were noted.

Leukocyte TL was measured by MMqPCR method as described by Cawthon [10] with minor modifications. Reference DNA at concentrations of 120, 40, 13.33, 4.44, 1.48 and 0.74 ng/μl were prepared from standard genomic DNA by serial dilution. Triplicates of reference DNA and DNA extracted from blood samples were taken in 1× master mix consisting of 0.5× SYBR Green I (Invitrogen, CA, USA); 0.5 U AmpliTaq Gold DNA polymerase (Applied Biosystems, CA, USA), 10 mM Tris-HCl, 50 mM KCl, 3 mM MgCl2 (Invitrogen), 0.2 mM each dNTP (MBI Fermentas, Hanover, MD,  USA), 1 mM DTT (Sigma, Germany), 1 M betaine (Sigma, Germany) and nuclease-free water in a final volume of 25 μl and added into reaction wells of 96-well PCR plate (Axygen, CA, USA) compatible with Real-Time PCR detection system (IQ5, software version 2.0, Bio-Rad Laboratories Inc., CA, USA) and amplified. One nontemplate control and two positive controls were also amplified in duplicates in each run. For the multiplex qPCR, HPLC-purified forward and reverse telomere primer pair (final concentration of 300 nM each) and single-copy gene, *albumin* primer pair (final concentration 300 nM each) were included in each reaction (Table 1). Thirty-five cycles of PCR were carried out as described in Figure 2 and signal was acquired using Bio-Rad iQ5 software version 2.0. The acquisition at 74°C provided the Ct values for telomeres and the acquisition at 88°C provided the cycle threshold (Ct) values for the single-copy gene (*albumin*). After the run was completed, the Ct values of telomere and *albumin* gene were transferred to spreadsheet separately to generate standard curve (Figure 3C & D) from which the copy number for telomere (T) and single-copy gene (S) values were generated. The ratio of the copy number of telomere (T) and the single-copy gene (S) was determined which is indicative of the average TL (i.e., the T/S ratio) per cell.

**Table 1.** **Primer sequence.**

**Telomere primers**	

Forward:	*ACACTAAGGGTTTGGGTTTGGGTTAGTGT*

Reverse:	*TGTTAGGTATCCTCATCCCTATCCCTATCCCTATCCCTAACA*

**Housekeeping gene, *albumin* primers**	

Forward:	*GGCGGCGGGCGCGGGCTGGGCGGAAATGCTGCACAGAATCCTTG*

Reverse:	*GCCCGGCCCGCCGCGCCCGTCCCGCCGGAAAAGCATGGTCGCCTGTT*

#### Quality control for MMqPCR assay

Two positive template control and one nontemplate control were included in each run. Intra- and interassay CV were assessed by ten replicates of one sample in the same batch and ten repeat analysis of one sample in different batches, respectively. We analyzed all samples in triplicate, and mean of two closest values was computed and if any sample showed >10% variation the sample was rejected and repeated in another experiment.

### Measurement of TRF length by Southern blot

**Figure 4.**
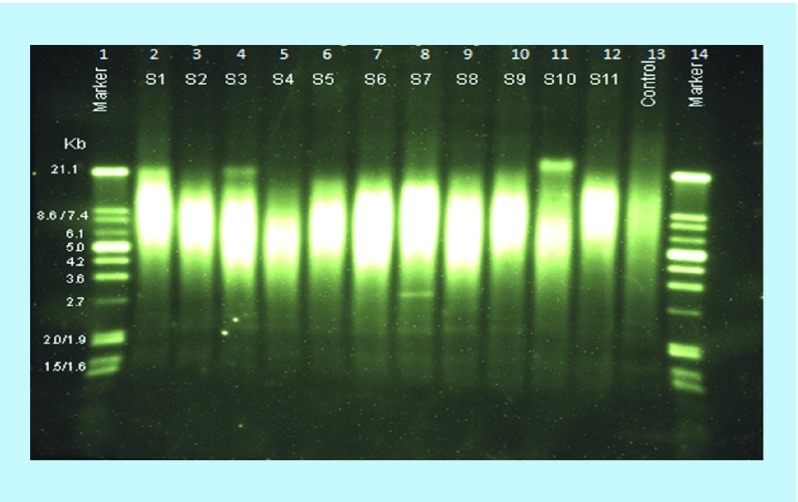
**Chemiluminescence detection of absolute telomere length by Southern blot.** Lane1, 14: Molecular marker; Lane 2–12: Unknown sample; Lane 13: Control. Telomeric restriction fragments were transferred to the nylone membrane and exposed to the imaging device using chemileumenescence detection system and telomeric restriction fragments were visualized. Telomere-restricted fragments length of each lane containing a different DNA sample was analyzed separately on the basis of migration distance of telomeric DNA in comparison to a known MW DNA marker. Bands in lane 4, 8 and 11 at the position 21, 2.7 and 21 kb, respectively are also telomeric restriction fragments.

TRF length was measured by Southern blot method, using the commercially available kit- teloTAGGG Telomere Length Assay Kit (catalog no: 12209136001, Roche, Germany) according to the manufacture instructions with minor modification as follows:

- Genomic DNA digestion and gel electrophoresis: genomic DNA isolated from the whole blood was used for Southern blot analysis. DNA concentration of samples was adjusted to 100 ng/μl. If DNA concentration was low, samples were lyophilized to bring the DNA concentration to 100 ng/μl. Genomic and control DNAs were digested with 1 μl of Hinf I/Rsa I enzyme mixture. Digested DNA was separated by 0.8% agarose gel electrophoresis along with digoxigenin (DIG) molecular weight marker. After gel electrophoresis, gel was prepared for blotting by depurination, denaturation and neutralization;
- Transfer of separated DNA to membrane: geparated DNA was transferred to positively charged nylon membrane. Equipment was setup for blotting in the following manner. Nylon membrane, 3 MM Whatman filter paper and blotting paper were cut into the same size as gel (12 × 15 cm^2^). In a plastic tray, 750 μl 20× saline-sodium citrate (SSC) buffer was taken and gel casting tray (12 × 15 cm^2^) was placed so as to create a platform in the buffer and a 3 MM Whatman filter paper sheet (15 × 20 cm^2^) was placed on the plane surface of the gel casting tray and fixed so that two sides of the sheet dipped properly in the 20× SSC buffer. Two Whatman filter papers (12 × 15 cm^2^) were placed onto the tray and air bubble was completely removed by rolling with a glass rod after wetting the paper properly with buffer. Gel was placed onto the sheet with the bottom side up and the nylon membrane was placed on the gel. Three sheets of 3 MM filter paper of the same size as nylon membrane were placed properly after removing the air bubbles. A bunch of blotting papers and then a glass plate were placed for smooth pressure followed by a 500 gm weight on the top. For maximum sensitivity and reproducibility, overnight blotting was performed. In the morning blotting membrane was placed in a UV crosslinker (DNA side facing upward). Transferred DNA was crosslinked to the membrane by 120 mJ UV light from a UV crosslinker;
- Hybridization and chemiluminescence detection of telomeric DNA: the crosslinked membrane was carefully placed in a hybridization bottle (DNA side facing inward) and incubated in freshly prepared and prewarmed 18 ml DIG-Easy-Hyb granules for 45 min at 42°C with gentle rotation in a hybridization oven. To prepare the hybridization solution, 1.2 μl of telomere probe was mixed in 6.5 ml fresh prewarmed DIG-Easy-Hyb granules. After completely removing the pregranules solution, hybridization solution was added to the membrane and incubated for 3 h at 42°C with gentle rotation in a hybridization oven. Hybridization solution was discarded and membrane was washed with stringent wash buffer I twice for 5 min at room temperature (15–25°C) and twice with stringent wash buffer II for 20 min at 50°C with gentle agitation in an incubator shaker. Membrane was washed with 100 ml 1× wash buffer for 3 min at room temperature with gentle agitation and then incubated with 100 ml blocking reagent for 30 min at room temperature. Blocking reagent was discarded and membrane was incubated with 75 ml anti-DIG-alkaline phosphatase for 30 min at room temperature with gentle agitation. Membrane was then washed twice with 100 ml of 1× wash buffer for 30 min with gentle agitation and after that the membrane was incubated with 100 ml of 1× detection buffer for 5 min at room temperature. The membrane was placed on an absorbent paper and immediately transferred to an opened hybridization bag with DNA side facing upward. Approximately 40 drops of substrate solution were spread carefully on the membrane without trapping the air bubbles in dark and incubated for 5 min at room temperature. Excess substrate solution was removed and the edges of bag were sealed using electric sealer. Membrane was exposed to the imaging device using chemiluminescence detection system and visualized image was saved (Figure 4). Telomeric-specific smear on the Southern blot image was converted into mean terminal restriction fragments length (TRF) with the help of Image J Software (version 1.43u, NIH, MD, USA). TRF length of each lane containing a different DNA sample was analyzed separately on the basis of migration distance of telomeric DNA in comparison to a known MW DNA marker.

## Statistical analysis

Linear regression was used to estimate the regression coefficient (r) between the two methods and scatter diagram was plotted using IBM SPSS statistics software version 22.0. Intraclass correlation coefficient was also calculated. For generating Bland–Altman plots, linear regression equation obtained from the scatter plot was used for converting T/S values generated in qPCR to kilobases. The regression equation used was Y = 1.89 × X + 5.46. Bland–Altman analysis was performed to assess bias and limits of agreement between the two methods as described by Gutierrez-Rodrigues *et al*. [16]. The average interassay CV was calculated by using the T/S ratio values of two samples repeated in assays on different days and for intra-assay CV samples repeated in same assay and was computed using the standard deviation (SD) divided by the overall mean and then multiplied by 100. We also computed CV for each pair of measures for each sample by calculating the SD between the pairs divided by mean and expressed as percentage. Arithmetic mean and median were calculated for the 94 pairs of CVs. Overall CV was calculated using the pooled mean and pooled SD of duplicate measures as reported by Pierce *et al*. [17].

## Results

The mean age of 94 participants was 43.2 years and mean BMI was 27.8, ±4.3 (range 19–41) kg/m^2^. 68.2% of participants were male. Figure 1 shows quality of the DNA obtained from peripheral blood. DNA quantity ranged from 80 to 120 ng/μl in the subjects. Figure 3 represents the parameters of real-time PCR experiments. The melt curve and melt peak show the homogeneity and specificity of amplified product of telomere and *albumin* (Figure 3A & B). Figure 3C and D show the standard curve used to determine the copy number of telomere (T) and *albumin* (S), respectively. In the semi log plot of DNA concentration versus cycle threshold, both curves were linear over the 81-fold DNA concentration range of standard DNA (R^2^ = 0.999 for both curve). The efficiency and slope of standard curves of telomere (E = 103.5%, slope = -3.241) and *albumin* (E = 102.9%, slope = -3.254) are acceptable. The amplification efficiency >110% is sign of primer-dimer formation and may be due to PCR inhibitors. The efficiency can be calculated using the equation: E = 10^(-1/slope)^-1. The coefficient of determination for both telomere as well as *albumin* standard curves was 0.999. A melt curve test at the end of thermocycling is also recommended for qPCR assay and tells the homogeneity of amplified product (Figure 3A). SYBR Green dye is a nonspecific dye that detects any dsDNA and nonspecific binding in a dissociation curve and gives multiple peaks. A melt peak represents the specificity of amplified product and gives a single peak of amplified product. The melt peak of our assay shows high specificity of amplified products of telomere and *albumin* (Figure 3B). No additional peak other than telomere and *albumin* means that there was no primer-dimer formation and nonspecific binding. These standard curves were used to determine relative T/S ratio or relative TL. The samples were run in triplicates and the mean of triplicates was used as final relative TL.

To examine the reproducibility of MMqPCR assay, the inter- and intra-assay CV% were assessed using the T/S ratio values. The average interassay CV was 5.3% when two samples were repeated in assays on different days and an intra-assay CV of 4.07% was observed when repeated in same assay. The arithmetic mean and median of CV calculated from pair-wise repeated samples were 6.99 and 4.04%, respectively. The overall/pooled CV was 9.06%.

TL (T/S ratio) ranged from 0.401 to 2.344 with mean values 1.02 (±0.32) in 94 subjects. Relative TL was significantly higher in females as compared with males. Figure 4 represents the chemiluminescence detection of absolute TL measured by Southern blot method. TL was measured on the basis of migration distance of telomeric DNA in the comparison with a known MW marker by using Image J software. The absolute TL was ranged between 5.59 and 10.05 kb. The inter- and intra-assay CV were 1.68% and 1.20% for Southern blot assay, respectively.

### Correlation of TL measured by qPCR with Southern blot

**Figure 5.**
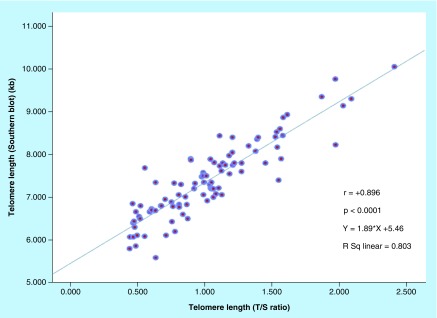
**Correlation of telomere length measured by monochrome multiplex quantitative polymerase chain reaction with Southern blot.** The scatter diagram comparing relative telomere length (telomeric DNA with fluorescent signals/single-copy housekeeping gene ratios) measured by monochrome multiplex quantitative polymerase chain reaction and absolute telomere length (kilobyte) measured by Southern blot analysis in leukocytes from 94 participants. A strong correlation was observed between both methods (r = +0.896).

**Figure 6.**
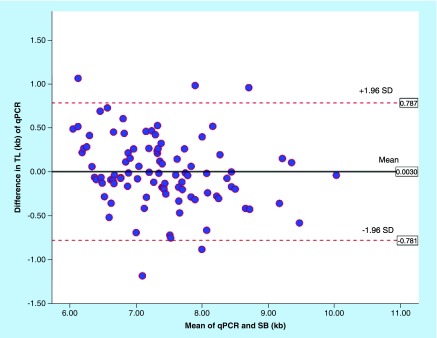
**Bland–Altman agreement between monochrome multiplex quantitative polymerase chain reaction (kilobyte) and Southern blot (kilobyte) analysis for leukocyte telomere length.** Bland–Altman plot for agreement analysis of quantitative polymerase chain reaction (kilobyte) and Southern blot analysis (kilobyte). The bias was 0.003 kb and limits of agreement ranged from -0.781 to 0.787 kb. qPCR: Quantitative polymerase chain reaction; SB: Southern blot; SD: Stansard deviation; TL: Telomere length.

Figure 5 shows the scatter diagram comparing TL measurement by Southern blot and MMqPCR. A significant positive correlation between TL measured by MMqPCR and Southern blotting was observed (R^2^ = 0.803, correlation coefficient r = +0.896, p < 0.0001). Intraclass correlation coefficient was 0.89 (95% CI: 0.84–0.93, p < 0.0001). Bland–Altman plot depicted in Figure 6 shows good agreement between the two methods. The mean difference (bias) between two methods was 0.003 kb with the limits of agreement between 0.787 and -0.781 kb.

## Discussion

The aim of this study was to validate the relative TL (T/S ratio) measured by MMqPCR by comparing with the gold standard absolute TL (kilobyte) measured by Southern blot technique. The results from our study showed that the relative TLs (T/S ratios) measured in peripheral leukocytes of 94 subjects by the MMqPCR assay correlated well with the absolute TL measured by Southern blot technique (r = +0.896 [R^2^ = 0.803], p < 0.0001). Some samples showed different length in MMqPCR and Southern blot assays, which may be due to the restriction enzymes used in Southern blot method for digesting genomic DNA which includes subtelomeric region with TRF, and polymorphism in this region impacts the final TL assessment; however, subtelomeric region is not amplified in the MMqPCR assay. Second, Southern blot technique may be unable to recognize the very short telomeres and ends lacking telomeres; however, very short telomeres can be amplified in qPCR assay.

The findings of our study are similar to that reported by others. In 2009, Cawthon RM [10] established the multiplex qPCR method and reported a good correlation with Southern blot method of TL measurement (R^2^ = 0.844). The same authors reported that the multiplex qPCR method is better correlated with Southern blot technique than singleplex qPCR method (R^2^ = 0.667) [9]. In the singleplex qPCR, telomere and single-copy gene are amplified in separate wells using equivalent volume of genetic material. Aviv *et al*. [18] also reported comparative analysis of leukocyte TL measured by qPCR and Southern blot assay, and found a significant correlation between both methods (r = 0.847, p < 0.0001). A measure of reliability of the measurements, intraclass correlation coefficient showed a good agreement between qPCR (kilobyte) and Southern blot assay (kilobyte) in our study. Bland–Altman analysis of our study also suggested a good agreement between both assays (bias ± SD = 0.003 ± 0.4, limits of agreement [LoA]: -0.781–0.787 kb). A 95% of the difference in the values between qPCR and Southern blot fell within ±2SD of mean difference. Gutierrez-Rodrigues *et al*. [16] carried out TL analysis by qPCR, Southern blot and flow-FISH assay in both healthy and in idiopathic pulmonary fibrosis/bone marrow failure patients and carried out Bland–Altman analysis. A good agreement between Southern blot and flow-FISH assay was observed in Bland–Altman analysis (bias ± SD = 0.17 ± 1.03, LoA: -1.88–2.24 kb for healthy subjects and bias ± SD = 0.00 ± 1.21, LoA: -2.41–2.41 for patients). Scatter plot showed a good correlation (R^2^ = 0.60 for healthy, R^2^ = 0.51 for patients) between the two methods. The authors, however, reported modest correlation between Southern blot and qPCR assay for the analysis of samples of healthy individuals (R^2^ = 0.3588) and low correlation for patients (R^2^ = 0.20) and Bland–Altman analysis showed poor agreement between Southern blot (kilobyte) and qPCR analysis (kilobyte) for both healthy controls (bias ± SD = 0.78 ± 1.34, LoA: -1.90–3.47 kb) and patients (bias ± SD = 1.15 ± 1.49, LoA: -1.84–4.14 kb). Between flow-FISH (kilobyte) and qPCR (kilobyte) also the agreement was poor for both healthy control (bias ± SD = -0.6 ± 1.27, LoA: -3.16–1.94 kb) and patients (bias ± SD = -1.15 ± 1.65, LoA = -4.45–2.15). The correlation was modest for healthy control (R^2^ = 0.337) and did not correlate for patient (R^2^ = 0.10) between flow-FISH (kilobyte) and qPCR (kilobyte) [16]. The authors reported a 15.9% interassay variability in qPCR assay. A recent study by Khincha *et al*. reported a moderate correlation between the qPCR and Southern blot method (R^2^ = 0.54 in dyskeratosis congenita patients and of R^2^ = 0.43 in unaffected relatives) [19].

Zanet *et al*. measured TL in peripheral blood mononuclear cell (PBMC), whole blood and dried blood spot (DBS) prepared from EDTA blood collected by venipuncture as well as from DBS prepared from finger prick by monoplex qPCR and multiplex qPCR. In this study TL in whole blood was less strongly correlated between monoplex qPCR and multiplex qPCR (R^2^ = 0.649). TL in DBS was strongly correlated with whole blood (R^2^ = 0.741) and PBMCs (R^2^ = 0.789) and mean TL was significantly higher in DBS [20]. Many studies have evaluated the source of variability in qPCR and attribute it to use of alternate thermal cycler instruments [15] and plate well positioning resulting in inconsistent temperature during amplification [21]. Hsieh *et al*. reported the optimization of MMqPCR conditions for relative TL on LightCycler 480 platform instead of Bio-Rad MyiQ PCR machine used by Cawthon for initial MMqPCR assay and was validated with Southern blot (R^2^ = 0.88) and flow-FISH assay (R^2^ = 0.81) [15]. The author used the commercial-purchased reaction mixture and optimized the conditions by varying the concentration of EDTA and MgCl_2_.

The amplification efficiency of qPCR can be determined by fitting a regression line to a subset of data point in a log-linear phase and is associated with bias in the analysis of qPCR data [21]. A standard curve is an excellent tool to assess the efficiency, precision and sensitivity of qPCR assay [22]. A good efficiency was reported in our study. Reproducibility is a concern with qPCR method as shown by a range of CVs (2–28%) reported in various studies [13,14]. The interassay CV was 5.3% and intra-assay CV was 4.07% in our study. A similar finding was reported by Cawthon RM (2009) with 5.22% intra-assay CV and 3.13% interassay CV for multiplex qPCR assay [10] and same author in 2002 reported a 5.8% interassay CV for monoplex qPCR [9]. An earlier study also reported an interassay CV of 6.45% for qPCR method [18]. However, some studies have reported a wide range of variability for qPCR assay; ranging 7–11% [23], 15–16.3% [14,16], 19% (intrabatch) and 28% (interbatch) [13] and 0.000–31.299% [24]. We modified the protocol described by Cawthon slightly [10]. We used a lower concentration of SYBR Green and Taq polymerase; and minimum optimized concentration of primers reducing the primer-dimer formation. All samples were analyzed in triplicate and mean of two closest values was computed, if any sample showed more than 10% variation the sample was repeated in another experiment. This may be the reason for better correlation observed in our study.

We found significantly higher TL in females which is in accordance with other studies [25,26]. Longer telomere in females is attributed to estrogen [27], which stimulates the activation of telomerase [25] and also has anti-inflammatory and antioxidant properties [28]. However, other studies have shown either no association [29] or longer telomere in males [30]. Variation in association between gender and TL could be due to different measurement methods, age group and cell types [31].

One limitation of qPCR assay is that it estimates a relative value of telomeric DNA per genome instead of absolute value of telomeric DNA represented in kilobyte. An author established an ‘absolute qPCR method’ to assess TL in kilobyte per diploid genome rather than relative values by comparing Ct values of unknown samples with artificially synthesized telomeric oligomers of known length as a reference [32]. However, this approach has been criticized for giving unrealistic values of TL when discussed among studies, suggesting these discrepancies could be due to variation in efficiencies between references oligomers and unknown samples [33].

## Conclusion & future perspective

In this comparative experimental study, qPCR method to measure relative TL was compared with the gold standard Southern blot technique that measures absolute telomere. A good correlation was observed between the two methods suggesting that MMqPCR method can be employed reliably for measurement of TL in epidemiological studies involving large number of samples. Since the sample size was small in the present study, a larger study with more samples spanning different ages so that a wider range of TL is covered would be of interest to assess correlation.

**Executive summary**

**Aim**

- To validate the results of telomere length measured by quantitative polymerase chain reaction (qPCR) with standard gold method Southern blot.

**Results & discussion**

- Relative telomere length was measured by monochrome multiplex qPCR method and absolute length of telomere restriction fragments by gold standard method, Southern blot, in leukocytes of healthy subjects and compared. A strong correlation was observed between both methods and reproducibility of the results from qPCR met acceptance criteria in interassay and intra-assay assessment.

**Conclusion**

- MMqPCR method can be employed reliably for measurement of telomere length in epidemiological studies involving large number of samples.
